# 

**Published:** 1838-01

**Authors:** 


### CONTENTS OF No. IX.

OF THE

### British and Foreign Medical Review.

JANUARY, 1838.

---

### Part First.—Analytical and Critical Reviews.

PAGE

Art. I.—1. Traité pratique des Maladies Vénériennes. Par H. M. J. Desruelles 1
A Practical Treatise on Venereal Diseases. By H. M. J. Desruelles, &c.
2. Practical Observations on the Venereal Disease, &c. By A. Colles, M.D. . ib.
3. Procès-verbaux des Séances tenues par les Médecins de Nantes, pour discuter la Valeur des Doctrines nouvelles, relativement à la Syphilis . . ib.
Minutes of the Proceedings held by the Medical Practitioners of Nantes, to discuss the Value of the new Doctrines relative to Syphilis.
4. Historisch-Kritische Darstellung des Streits über die Einheit oder Mehrheit der Venerischen Contagien. Von Dr. Friedrich Oesterlen . . ib.
Historical and Critical Display of the Controversy on the Unity or Plurality of the Venereal Contagion. By Dr. F. Oesterlen.
5. Die Mercurialkrankheit in allen ihren Formen, &c. Von G. L. Dieterich, M.D. ib.
The Mercurial Disease in all its Forms, &c. By G. L. Dieterich, M.D.
6. Die Behandlung der Lustseuche ohne Quecksilber, &c. Von Dr. Oppenheim 2
The Treatment of Syphilis without Mercury, &c. By Dr. Oppenheim.
7. Traité pratique de la Syphilis. Par Baron Philip Boyer . . ib.
A Practical Treatise on Syphilis. By Baron Philip Boyer.
8. Practical Treatise on Urethritis and Syphilis. By Wm. Henry Judd, M.R.C.S. ib.
9. Treatise on the Venereal Disease. By John Hunter, F.R.S., &c. . ib.

Art. II.—Lehre von den Augenkrankheiten. Von Dr. Anton Rosas . 31
The Study of the Diseases of the Eye. By Dr. Anton Rosas.
2. Dell' Ottalmoscopia, &c. Del Dottore Luigi Marchetti da Cremia . ib.
A Dissertation on Ophthalmoscopy, &c. By Dr. Marchetti.
3. De l'Emploie de l'Excision et de la Cauterisation dans l'Ophthalmie blennorrhagique. Par E. F. Julliard . . . . . . 31
Use of Excision and Cauterization in purulent Ophthalmia. By E. F. Julliard.
4. The Cyclopædia of Practical Surgery. Edited by W. B. Costello, M.D. Part I. Art. Amaurosis, by Frederick Tyrrell, Esq. . . . ib.
5. A Manual of the Diseases of the Eye. By S. Littell, Jun. M.D. . . 31

Art. III.—The Works of John Hunter, F.R.S.; with Notes. Ed. by J. F. Palmer 46
Lectures on the Principles of Surgery; with Notes. By J. F. Palmer.

Art. IV.—Essai sur la Colique de Plomb. Par Augustin Grisolle, M.D. . 55
Essay on the Colic produced by Lead. By Augustin Grisolle, M.D.

Art. V.—What Asylums were, are, and ought to be. By W. A. F. Browne . 65

Art. VI.—1. Handbuch der Physiologie des Menschen. Von Dr. Müller . 75
Manual of Human Physiology. By Dr. Johannes Müller.
2. Elements of Physiology, by J. Müller. Translated by Wm. Baly, M.R.C.S. . . ib.
3. Die Erscheinungen und Gesetze des lebenden menschlichen Körpers im gesunden und kranken Zustande. Von Dr. Fr. Arnold und Dr. J. W. Arnold . ib.
The Phenomena and Laws of the living Human Body, in Health and in Disease.
4. Rudiments of Physiology. By John Fletcher, M.D. . . . ib.
5. Outlines of Human Physiology. By Herbert Mayo, F.R.S. . . ib
6. [illegible]uman Physiology. By Robley Dunglison, M.D. . . . ib.

Art. VII.—Practical Essay on Beriberi, and Rheumatism. By Mr. Malcolmson 116

Art. VIII.—Ueber Paralyse der Inspirations-Muskeln. Von Dr. L. Stromeyer 133

On Paralysis of the Muscles of Inspiration. By Dr. Louis Stromeyer.

a

4282

PAGE

ART. IX.—On the Nature and Treatment of the Diseases of the Heart; with some new Views on the Physiology of the Circulation. By JAMES WARDROP, M.D. 146

ART. X.—Mémoires de la Société Médicale d'Observation, de Paris. Tome I. . 154
Memoirs of the Medical Society of Observation, of Paris. Vol. I.—M. LOUIS' Numerical Method.

ART. XI.—An Experimental Essay on the relative Physiological and Medicinal Properties of Iodine and its Compounds. By CHARLES COGSWELL, A.B. M.D. 162

ART. XII.—Memorie della Società Medico-Chirurgica di Bologna. Vol. I. Fasc. 1, 2 167

Memoirs of the Medico-Chirurgical Society of Bologna. Vol. I. Fasc. 1 and 2.

ART. XIII.—A Medico-legal Treatise on Homicide by external Violence. By ALEXANDER WATSON, F.R.C.S.E. . . . . 171

ART. XIV.—Beobachtungen auf dem Gebiete der Pathologie und Pathologischen Anatomie. Gesammelt von Dr. JOH. FRIED. HERM. ALBERS. Erster Theil 181
Observations in the Department of Pathology and Pathological Anatomy. Collected by Dr. ALBERS. Vol. I.

ART. XV.—Essay on Pyrexia, or Symptomatic Fever. By H. CLUTTERBUCK, M.D. 185

ART. XVI.—1. Die Pest des Orients. Von Dr. C. J. LORINSER . . 191
The Oriental Plague; its Cause, and Means of preventing it. By Dr. LORINSER.

2. De Peste Antoniniana Commentatio. Scripsit JUST. FRID. CAR. HECKER, M.D. ib.
Essay on the Plague which prevailed in the Reign of Antoninus. By Dr. HECKER.

ART. XVII.—On a new Mode of Treatment employed in the Cure of various Forms of Ulcer and granulating Wounds. By F. C. SKEY, F.R.S. . . 195

ART. XVIII.—First Principles of Medicine. By ARCHIBALD BILLING, M.D. A.M. 200

ART. XIX.—Elémens de Zoologie; ou Leçons sur l'Anatomie, la Physiologie, la Classification, et les Mœurs des Animaux. By M. H. MILNE-EDWARDS, M.D. 204

Elements of Zoology; or Lectures on the Anatomy, Physiology, Classification, and Habits of Animals. By Dr. M. H. MILNE-EDWARDS.

ART. XX.—The Teeth a Test of Age. By EDWIN SAUNDERS . . 205

ART. XXI.—Bidrag til Bornekoppernes og Vaccinationens Historie i Danmark. Af JOH. CHR. W. WENDT, M.D. . . . . 207
Contributions to the History of the Small-pox and of Vaccination in Denmark. By J. C. WENDT, M.D.

---

### PART SECOND.—Bibliographical Notices.

ART. I.—Transactions of the Philosophical and Literary Society of Leeds. Vol. I. 211

ART. II.—Die Gerichtlich-chemischen Untersuchungen. Vom Dr. C. GUSSEROW 212

Researches in Medico-legal Chemistry. By Dr. C. GUSSEROW.

ART. III.—Treatise on the Influenza of 1837. By P. BLAKISTON, M.A. . 214

ART. IV.—On the Use of Auscultation and Percussion in the Diagnosis of Diseases of the Organs of Respiration and Circulation. By JULIUS WOLFF, M.D. . 215

ART. V.—Hints to Mothers for the Management of Health. By T. BULL, M.D. . ib.

ART. VI.—Nature and Treatment of Diseases of the Ear. By Dr. WM. KRAMER. Translated from the German, by J. R. BENNETT, M.D. . . 216

ART. VII.—Elements of Anatomy. By JONES QUAIN, M.D. . . ib.

ART. VIII.—Elements of Chemistry. By the late EDWARD TURNER, M.D. . ib.

### Part Third.—Selections from Foreign Journals.

### ANATOMY AND PHYSIOLOGY.

PAGE
On the Anatomy of the Eyelids. By Dr. Zeis, of Dresden . . . 217
Microscopical Researches on the ultimate Structure of the Nerves. By Dr. Berres 219
On the Microscopic Structure of the Brain. By Dr. Luigi Calori . . 220
On the Fibrous Membrane beneath the Pleura Pulmonalis. By M. Bazin . ib.
Examination of the Bodies embalmed by Signor Tranchina . . 221
On the Action of Strychnia on the Nervous System. By Dr. H. Stannius . ib.
Experiments on Animals with the Blood of Cholera Patients. By Dr. Namias . 223
Experimental Examination into the Opinions of Sir C. Bell relative to the anatomical and physiological Characters of the Spinal Marrow. By Dr. H. Smith 224

### PATHOLOGY, PRACTICAL MEDICINE, AND THERAPEUTICS.

Different Views of the Nature and Treatment of Tetanus: 1, by Signor Morotti Francesco, of Vercelli; 2, by Dr. Bruggemann, of Magdeburg; 3, by Dr. Ferrus; 4, by Dr. Smith, of Port au Prince; 5, by Dr. Henry 226–230
On the present predominant Views regarding Acute Rheumatism. By Dr. Zeroni 230
On the Cause, Seat, and Nature of the Asiatic Cholera. By Dr. Bellingeri . 234
Cases of severe Lesion of the Brain: 1, by G. W. Boerstler, of Lancaster, Ohio; 2, by M. Bouvier . . . . . . 236–8
Efficacy of Sugar of Lead in Fever with Ulcers of the Intestines. By Prof. Nasse 239
Moral Management of the Insane in America . . . . . ib.
Treatment of Delirium Tremens by Digitalis and Opium, by Drs. Huss and Weisse 242
New Mode of treating Empyema. By Dr. Reybard . . . ib.
Case of Dyscrasy, treated with Sugar. By Professor Dunglison . . 243
Curious Case of Absorption of Bone. By Dr. John H. Marable, of Tennesse 244

### SURGERY.

On the Treatment of Erysipelas by raw Cotton. By M. Reynaud . . 244
Rhinoplastic Operation. By Dr. Warren, of Boston . . . 245
The Causes which retard or prevent the Consolidation of Fractures. By M. Fleury 248
Progress of Lithotrity: 1, by Dr. Martins; 2, by M. Civiale; 3, by M. Ségalas 249
On the Separation of the Epiphyses of Bones. By M. Roux de Brignolle . 250
On the Effects of Pus in Contact with Osseous Tissue. By M. Lagémard . 251
On the external Use of Calomel in Inflammation of the Eyes. By Dr. Fricke . ib.
Account of a new Cupping-glass. By M. Lafargue . . . 252
Case of a Silver Teaspoon swallowed during a Paroxysm of Madness. By Dr. Otto ib.
Ossification of the Vitreous Humour. By Professor Grillo . . 253
Extraction of a foreign Body from the Female Bladder. By M. Thomas . ib.

### MIDWIFERY.

On Extra-uterine Pregnancy. By Dr. Dezeimeris . . . 254
Unconscious Delivery. By M. Leonhard . . . . 255
Speculum Uteri made of Glass . . . . . . ib.
Instances of Menstruation occurring in Old Age . . . . 256
Rupture of the Cæcum during Labour. By Dr. Stampf, of Stargard . . ib.
Vagitus Uterinus before the Rupture of the Membranes . . . 257
Injections into the Vessels of the Umbilical Cord, in Cases of retained Placenta . ib.

### HYGIÈNE.

Diseases of Cutlers. By M. Chevallier . . . . . ib.

### MEDICAL STATISTICS.

On the Prevalence and Mortality of Cholera in Venice, during the Years 1835-6 . 259
Statistics of Cholera in Naples . . . . . . 260
Sydney Dispensary, New South Wales . . . . . 261
Contributions to the Statistics of Diseases. By Dr. Casper . . 262

PAGE

VETERINARY MEDICINE.

Observations on Rabies in Animals. By Dr. WAGNER . . . 262

ANIMAL CHEMISTRY.

Microscopical Observations relative to Stains upon Linen. By M. RATTIER . 266
Observations on Blue Urine. By M. DRANTZ . . . . ib.

---

### PART FOURTH.—Selections from British Journals.

PHYSIOLOGY.

On the Acoustic Principles of the Stethoscope. By Dr. COWAN . . 267
Reaction of Saliva upon red and blue Litmus and Turmeric Papers. By Mr. LAYCOCK 268
Observations on the Pulses of Infants. By Mr. GORHAM . . ib.

PATHOLOGY, PRACTICAL MEDICINE, AND THERAPEUTICS.

Report of St. John's Fever and Lock Hospitals, Limerick. By Dr. GEARY . 270
On the Inadequacy of the Valves of the Aorta. By Dr. HENDERSON . . 272
Effects of deficient Ossification of the Cranium. By Mr. GRANTHAM . . 273
On Aortitis, as one of the Causes of Angina Pectoris. By Dr. CORRIGAN . ib.
On the Remedial Powers of the Aconitum Napellus in Headach. By Mr. RADLEY 275
Use of Tartar Emetic and Opium in Spasmodic Affections. By Mr. ACKERLEY . ib.
Observations on the Nature and Treatment of Neuralgia. By Dr. OSBORNE . 276
On artificial Respiration in the Asphyxia of Still-born Children. By Dr. CAPE . 277
Disease of the Spino-occipital Articulation. By Mr. BLACKLEY . . 278
On Acupuncture in Hydrocele and Ascites. By Messrs. LEWIS and KING . 279
Treatment of Spasmodic Cholera by Sugar of Lead and Opium. By Dr. GRAVES . 280
On the Use of Colchicum in Scarlatina. By Mr. TAIT . . . 281

SURGERY.

Case of Partial Paralysis of the Face from Injury of the Head. By Mr. SHAW . 281
Undescribed Displacement of Bones of the Fore-arm in Children. By Mr. GARDNER 284
Objections made to the Performance of Extraction in Cataract. By Dr. KENNEDY . 285
On the Division of the Tendo-Achillis in Cases of Club-foot. By Mr. WHIPPLE . ib.
Use of Nitrate of Silver in some Cutaneous Diseases. By Mr. CHAPMAN . 287
Cure of congenital Club-foot by Division of the Tendo-Achilis. By Dr. INGLIS . 288

MIDWIFERY.

Cases of Inversion of the Uterus; with Remarks. By Mr. RADFORD . . 288

Case of Hidrosis, or Hidrotic Fever; with Remarks. By Mr. LEVER . . 290

MEDICAL STATISTICS.

Statistics of the Sickness and Mortality which occurred among the Troops employed on the Expedition to the Scheldt, in the Year 1809. By Mr. MARSHALL . 290
Of the Probability of Death and Recovery in Asiatic Cholera. By Mr. FARR . 292

---

### PART FIFTH.—Medical Intelligence.

Progress of the Anatomy and Physiology of the Nervous System during the Year 1836, by Professor MÜLLER, of Berlin . . . . 293
Report on the Radical Cure of Hernia by means of Trusses with solid Blocks, (Pads,) by Drs. COATES and PARRISH . . . . 300
Improved Female Syringe, by Dr. CHASE . . . . 307
Abstract of an Experimental Investigation into the Functions of the Eighth Pair of Nerves. By JOHN REID, M.D. . . . . . 308
Medical Relief under the new Poor-Law . . . . 310
Medical Certificates to Assurance Societies . . . . 311

OBITUARY: Dr. JOHN MACKINTOSH, of Edinburgh . . . ib.

Dr. WILLIAM WEST, of Dublin . . . . 313

